# Mitotherapy attenuates sepsis-induced brain injury in rats subjected to cecal ligation and puncture: Role of SIRT-1/PGC-1α network

**DOI:** 10.22038/ijbms.2025.84848.18363

**Published:** 2025

**Authors:** Behnaz Mokhtari, Alireza Alihemmati, Soleyman Bafadam, Solmaz Boraghi, Reza Badalzadeh, Ata Mahmoodpoor

**Affiliations:** 1 Drug Applied Research Center, Tabriz University of Medical Sciences, Tabriz, Iran; 2 Molecular Medicine Research Center, Tabriz University of Medical Sciences, Tabriz, Iran; 3 Department of Physiology, Faculty of Medicine, Tabriz University of Medical Sciences, Tabriz, Iran; 4 Department of Anesthesiology, Faculty of Medicine, Tabriz Azad Medical University, Tabriz, Iran; 5 Evidence-Based Medicine Research Center, Tabriz University of Medical Sciences, Tabriz, Iran

**Keywords:** Brain, Mitochondria, Mitotherapy, Neuroprotection, Sepsis

## Abstract

**Objective(s):**

Sepsis-induced brain injury poses a critical challenge with limited therapeutic options. Mitochondrial dysfunction is a central contributor to this pathogenesis. The current work aimed to examine the effects of mitochondrial transplantation, termed “mitotherapy”, on sepsis-induced brain injury using a cecal ligation and puncture (CLP) rat model.

**Materials and Methods:**

Male Wistar rats (n=40, 12 weeks old, weighing 250–300 g) were allocated into groups with or without CLP-induced sepsis, receiving mitotherapy via single or two repetitive injections post-CLP. In recipient groups, mitochondria harvested from donor rats were injected intravenously (400 μl of mitochondrial suspension containing 7.5×10^6^ mitochondria/ml of respiration buffer). Twenty-four hours post-operation, the behavioral phenotype was tested by using the Murine Sepsis Score (MSS). Brain morphological examination was conducted using Hematoxylin and Eosin staining. Mitochondrial function was measured by evaluating membrane potential, reactive oxygen species production, and adenosine triphosphate content. The expression of genes regulating mitochondrial biogenesis (SIRT-1, PGC-1α) and fission/fusion (Drp1, Mfn1, Mfn2) was determined via real-time polymerase chain reaction. The levels of inflammatory cytokines (TNF-α, IL-1β, IL-6) were measured using Enzyme-Linked Immunosorbent Assay.

**Results:**

Mitotherapy reduced MSS and alleviated histopathological changes associated with sepsis-induced brain injury. Furthermore, it restored mitochondrial functional indices, up-regulated genes involved in mitochondrial biogenesis and fusion, and reduced inflammatory cytokine levels (*P*<0.05). Repetitive injections provided greater therapeutic benefits than a single injection.

**Conclusion:**

Mitotherapy mitigated sepsis-induced brain injury by improving mitochondrial function, biogenesis, and dynamics within the SIRT-1/PGC-1α network and concurrently suppressing inflammation. Repetitive injections exhibited enhanced potency, suggesting a novel avenue for managing sepsis-associated brain dysfunction.

## Introduction

Sepsis is a life-threatening global healthcare challenge characterized by organ dysfunction resulting from a dysregulated host response to infection (1). Among the organs affected, the brain is particularly vulnerable, often exhibiting the earliest signs of dysfunction clinically identified as sepsis-associated encephalopathy (SAE) (2). SAE manifests as a spectrum of neurological impairments, ranging from confusion to coma, and is associated with structural brain changes, including gray and white matter atrophy, persistent cognitive deficits, and increased mortality (3). The significant morbidity and mortality associated with SAE underscore the urgent need to better understand its underlying mechanisms and develop targeted therapeutic interventions. Current treatment strategies for sepsis and SAE remain largely supportive, focusing on infection control and organ stabilization, but they often fail to address the root causes of brain injury. This limitation highlights the critical importance of exploring innovative approaches that target the molecular and cellular pathways driving sepsis-induced brain dysfunction. By advancing our understanding of these mechanisms, we can pave the way for novel therapies that improve outcomes for patients with sepsis and SAE (4).

A growing body of evidence highlights the critical roles of mitochondrial dysfunction and the inflammatory response in driving sepsis-induced brain injury (5, 6). During sepsis, inflammatory cytokines from the innate immune response induce mitochondrial permeability transition, suppress oxidative phosphorylation, and reduce adenosine triphosphate (ATP) production. Concurrently, they increase reactive oxygen species (ROS) generation and disrupt signaling pathways essential for mitochondrial biogenesis and dynamics (7). These dysregulations in mitochondrial quality control mechanisms impair cellular energy metabolism, contributing to the brain dysfunction and failure commonly observed in septic patients (8, 9). While it remains debated whether mitochondrial dysfunction is a cause or consequence of sepsis, its central role in perpetuating a destructive cycle of inflammation, oxidative stress, and cellular damage is well-established (10). This cycle exacerbates organ dysfunction and worsens patient outcomes, making mitochondrial function a promising therapeutic target for sepsis management. Despite a consensus that enhancing mitochondrial quality control mechanisms can alleviate sepsis-induced organ dysfunction, clinical translation remains limited due to a lack of effective diagnostic tools and targeted therapies (11-13). Addressing these gaps is essential for developing innovative treatments that improve outcomes in sepsis and its associated complications.

Mitochondria-targeting treatments have shown promise in improving mitochondrial function and alleviating symptoms of mitochondrial dysfunction in sepsis. However, their efficacy is often limited by irreversible changes, such as mitochondrial DNA mutations, that occur during sepsis (14). Mitochondrial transplantation (mitotherapy) has emerged as a groundbreaking approach to overcome these limitations. By transferring healthy exogenous mitochondria into cells with defective mitochondria, mitotherapy aims to restore cellular function and prevent further damage (15). Preclinical studies in experimental sepsis models have demonstrated its therapeutic potential. For instance, in a mouse model of sepsis, mitotherapy shifted microglial polarization from the pro-inflammatory M1 to the anti-inflammatory M2 phenotype, resulting in neuroprotection and improved cognitive outcomes (16). Similarly, in a rat polymicrobial sepsis model, mitotherapy enhanced survival, improved bacterial clearance, and attenuated mitochondrial dysfunction and apoptosis in septic spleens (17). Another study showed that mitotherapy reduces systemic inflammation, mitigates organ injury, and improves survival in septic mice (18). Our previous work demonstrated that mitotherapy improved 72-hr survival and protected against sepsis-induced myocardial dysfunction in a rat cecal ligation and puncture (CLP) model. These protective effects were mediated by enhanced mitochondrial function, biogenesis and dynamics promotion, and inflammatory response suppression (19). These findings suggest that mitotherapy may have the potential to address the pathological changes induced by sepsis in the brain, although the underlying mechanisms remain to be fully elucidated.

Given the central role of mitochondrial dysfunction in sepsis-induced brain injury and the emerging therapeutic potential of mitotherapy, we hypothesized that mitotherapy could offer significant neuroprotective benefits in this context. To test this hypothesis, we investigated the effects of mitotherapy on brain injury using a rat CLP-induced sepsis model, with a particular focus on mitochondrial mechanisms. This study aims to provide novel insights into the therapeutic potential of mitotherapy for mitigating sepsis-induced brain injury, offering a foundation for future translational research in this critical area.

## Materials and Methods

### Experimental animals and ethical considerations

Adult male Wistar rats (n = 40), aged 12 weeks and weighing 250–300 g, were employed for experimental grouping. Additionally, young male Wistar rats (n = 10), aged 8 weeks and weighing 180–200 g, were used as donor rats to harvest isolated mitochondria. These rats were procured from the animal center at Tabriz University of Medical Sciences. The rats were housed in cages maintained in a pathogen-free environment within a designated animal room. They were subjected to a regulated light-dark schedule of 12 hr each, with the room temperature set at 25 ± 2 °C and humidity at 55%. The rats were provided free access to standard chow and water, and a 7-day acclimatization period was permitted before the experimental interventions. All animal experimental procedures were conducted strictly following the guidelines outlined in the 8^th^ Edition of the Guide for the Care and Use of Laboratory Animals, published by the US National Institutes of Health (National Research Council 2011). The study protocol received ethical validation from the Ethics Board at Tabriz University of Medical Sciences, Tabriz, Iran (Ethics Approval Number: IR.TBZMED.VCR.REC.1399.111). 

### In vivo experimental setup

Using a random number table, rats were distributed into four experimental groups. In each group, there were 10 rats utilized for Murine Sepsis Score (MSS) assessment, along with subsequent histopathological and molecular analyses, as outlined below:

Sham: Rats underwent an incision and suturing of the abdominal cavity without CLP; Sepsis: Rats underwent CLP operation; Sepsis+Mito1: Rats underwent CLP operation and were intravenously administered a 400 μl mitochondrial suspension, comprising 7.5 × 10^6^ mitochondria/ml of respiration buffer, precisely one hour after the CLP surgery (19); and Sepsis+Mito2: rats underwent CLP operation and received repeated intravenous administrations of isolated mitochondria at the 1-hr and 7-hr time points after the CLP surgery (19).

It is important to note that the Sham and Sepsis groups received a respiration buffer without isolated mitochondria as vehicle control. Twenty-four hours after the sham or CLP operation, an MSS assessment was conducted on ten rats from each group. Following the MSS assessment, four rats from each group were randomly selected for histopathological examination, while the remaining rats were allocated for molecular analyses. For euthanasia, the rats were handled in strict accordance with the animal care guidelines established by Tabriz University of Medical Sciences (Tabriz, Iran). The brain was carefully extracted from the skull, and the prefrontal cortex was dissected using precise anatomical landmarks as a guide. The dissected prefrontal cortex tissue was then processed further for subsequent analyses, as required by the specific objectives of this study.

### Procedure for mitochondria isolation and transplantation

In summary, donor rats were subjected to anesthesia through an intraperitoneal injection of a mixture containing ketamine (60 mg/kg) and xylazine (10 mg/kg). The pectoralis major muscle sample was procured using a biopsy punch. The muscle sample, weighing around 40 mg, was dissected and then homogenized in an isolation buffer under ice-cold conditions at a temperature of 4 °C. This buffer consisted of 2 mmol ethylenediaminetetraacetic acid, 10 mmol 4-(2-hydroxyethyl)-1-piperazineethanesulfonic acid (HEPES), 70 mmol sucrose, and 200 mmol mannitol (pH 7.4). The homogenization process was carried out using a pre-cooled 2.0 ml Dounce Homogenizer, with approximately 1 ml of buffer per 15 mg of tissue. The centrifugation process involved two steps: first at 1,200 × g for ten minutes to separate the supernatant from the homogenates, followed by a second step at 12,000 × g for ten minutes to yield the mitochondrial pellet. The mitochondrial pellet was suspended in a 100 µl of respiration buffer consisting of 0.08 mmol adenosine diphosphate, 1 mmol ATP, 1 mmol dithiothreitol, 2 mmol dipotassium phosphate, 5 mmol sodium succinate, 10 mmol HEPES, and 250 mmol sucrose (pH 7.4), and subsequently employed for transplantation (20). The protein concentration was quantified using the bicinchoninic acid method, meticulously adhering to the manufacturer’s provided instructions (Sigma-Aldrich, USA), with bovine serum albumin employed as a reference standard. The hemocytometry method was employed to quantify the number of isolated mitochondria (21). Mitochondrial transplantation involved injecting 400 μl of mitochondrial suspension containing 7.5 × 10^6^ mitochondria/ml of respiration buffer via the tail vein over two minutes.

### Establishment of a sepsis rat model

This study mimicked sepsis conditions seen in clinical settings using the CLP model, which entails surgery to breach the gut barrier. The rats were allowed unlimited access to water during their 12-hr fast before the procedure. Following this, the rats underwent anesthesia through an intraperitoneal injection of a mixture containing ketamine (60 mg/kg) and xylazine (10 mg/kg), creating a midline incision (2–3 cm in length) on the abdomen under sterile conditions allowed for the isolation and exposure of the cecum. After securely ligating the cecum just beneath the ileocecal valve, it underwent two perforations using an 18-gauge needle. Subsequently, gentle pressure was applied to the cecum to facilitate the expulsion of a small quantity of fecal matter into the abdominal cavity, confirming the effectiveness of the punctures. Once the cecum was placed back within the peritoneal cavity, the incision was sealed using a sterile 5–0 silk suture. In the sham-operated group, laparotomy was performed, and the cecum was exposed as described earlier but without ligation or perforation. Immediately following the surgical procedure, all animals received a subcutaneous injection of 5 ml/100 g isotonic saline solution (0.9% NaCl, 37°C), administered at five different sites to ensure proper distribution and minimize the risk of localized edema. A second dose of saline was administered 12 hr post-surgery. Upon awakening, the rats were provided unrestricted water access but were deprived of food. Antibiotics were administered subcutaneously at 6-hr intervals, with ceftriaxone at 30 mg/kg and clindamycin at 25 mg/kg (22, 23). 

### Assessment of MSS

Twenty-four hours post-sham or CLP surgery, groups of ten rats each underwent behavioral phenotype testing using the MSS (24). Seven variables were assessed: (1) physical appearance, (2) degree of consciousness, (3) activity, (4) reaction to stimuli, (5) eye condition, (6) respiration rate, and (7) respiration quality. Each variable was scored from 0 to 4, and two independent investigators, blinded to the experimental groups, independently performed the scoring. The final score was the average of their evaluations. The severity of sepsis in each rat was determined using the average score, with higher scores indicating a greater level of severity. After completing the MSS assessment, four rats from each group were selected for histopathological examination, and the remaining rats were designated for molecular evaluations.

### Evaluation of brain histopathological changes

After completing the MSS assessment, the rats were anesthetized via intraperitoneal injection of a mixture containing ketamine (60 mg/kg) and xylazine (10 mg/kg). They then underwent cardiac perfusion with a cooled physiological saline solution (0.9% NaCl) to remove blood and debris from the brain tissue. This was immediately followed by perfusion with 4% paraformaldehyde (Sigma-Aldrich, USA) in phosphate-buffered saline to ensure proper fixation. The prefrontal cortex was dissected and post-fixed in 4% paraformaldehyde for 24 hr at 4 °C and then embedded in paraffin. For morphological analysis, paraffin-embedded tissues were sectioned into 5-µm thick slices using a microtome and subjected to Hematoxylin and Eosin staining. A blinded investigator examined the stained sections under a light microscope (Olympus, Tokyo, Japan). Four randomly selected microscopic fields per tissue section were evaluated for histopathological changes. The severity of these changes was semi-quantitatively scored as follows (25):

**(-)**: No histological changes detected in any field.

**(+)**: Histological changes detected in one field.

**(++)**: Histological changes detected in two fields.

**(+++)**: Histological changes detected in three fields.

**(++++)**: Histological changes detected in all four fields.

### Analysis of mitochondrial functional indices

*Brain mitochondria isolation*

To assess mitochondrial functional indices, including mitochondrial membrane potential, ROS production, and ATP levels, mitochondria were isolated from the brains of rats using the method detailed in the “Procedure for mitochondria isolation and transplantation” section. In brief, brain samples were homogenized in an ice-cold isolation buffer (composition specified in the referenced section). The homogenate was first centrifuged at 1,200 × g for ten minutes at 4 °C to remove cellular debris and nuclei. The supernatant was centrifuged at 12,000 × g for ten minutes at 4 °C to pellet the mitochondria. The resulting mitochondrial pellet was gently resuspended in respiration buffer (composition specified in the referenced section) and maintained on ice to evaluate mitochondrial membrane potential, ROS production, and ATP levels, as detailed in the corresponding sections (20).

*Mitochondrial membrane potential*

The procedure for evaluating the mitochondrial membrane potential in brain tissue samples involved utilizing a ﬂuorescent dye 5,5’,6,6’-tetrachloro-1,1’,3,3’-tetraethylbenzimidazolylcarbocyanine iodide (JC-1). The assessment was carried out according to the instructions provided by the mitochondrial membrane potential assay kit (Sigma-Aldrich, USA). The brain specimens were sliced into 10–14 μm thick sections using a cryostat at -18 °C. Subsequently, the sections were treated with JC-1 dye (20 µmol/ml in phosphate-buffered saline) and examined using a fluorescence microscope. JC-1 dye accumulates in the mitochondria in a potential-dependent manner. JC-1 aggregates, which emit red fluorescent in healthy cells, were exited at 525 nm and detected at an emission wavelength of 590 nm. Conversely, JC-1 monomers, emitting green fluorescence in unhealthy cells, were exited at 490 nm and detected at an emission wavelength of 530 nm. Fluorescent intensity measurements were taken using a spectrofluorometer (FP750), and the shifts in mitochondrial membrane potential were computed based on the ratio between red and green ﬂuorescence intensities. A decline in the red to green ﬂuorescence intensity ratio signifies mitochondrial membrane depolarization. The data was presented in the form of ﬂuorescence units per microgram of protein.

*Mitochondrial ROS production*

To assess mitochondrial ROS production, the mitochondrial pellets were subjected to a 30-minute incubation with a 2 µM solution of 2′,7′-dichlorofluorescein diacetate (DCFDA) dye under room temperature. DCFDA, a dye with ﬂuorescence-inducing properties, quantifies the activity of peroxyl, hydroxyl, and other ROS within cellular and organelle contexts. After diffusion into the organelle, ROS trigger the oxidation of DCFDA, leading to the creation of an intensely fluorescent substance identified as 2’,7’-dichlorofluorescein (DCF). The fluorescence emitted by DCF was identified using a fluorometric technique, with excitation and emission wavelengths set at 480 nm and 530 nm, respectively. Elevated fluorescent intensity corresponded to a rise in mitochondrial ROS production. The fluorescence intensity per milligram of protein in the samples was utilized to report the levels of mitochondrial ROS.

*Mitochondrial ATP levels*

The measurement of mitochondrial ATP content was carried out utilizing an associated bioluminescent assay kit (MAK190, Sigma-Aldrich, USA) as per the manufacturer’s guidelines. To summarize, 10 mg brain tissue samples were lysed in 100 μl of ATP assay buffer. Following this, the ATP probe was introduced along with the developer (supplied in the kit), and the solution’s absorbance was measured at 570 nm. The samples’ ATP levels were presented as nmol/mg protein.

### Analysis of gene expression

Real-time polymerase chain reaction (PCR) was used to evaluate the expression of SIRT-1, PGC-1α, Drp1, Mfn1, and Mfn2 genes. To begin with, approximately 100 mg of fresh prefrontal cortex tissues, which had been treated with RNase Later solution, underwent RNA extraction using the Trizol method as per the guidelines provided by the manufacturer (Roche, Germany). The RNA yield and purity were determined using a NanoDrop ND-2000C spectrophotometer (Thermo Fisher Scientific, USA) at 260/280 nm wavelength. An Exiqon complementary DNA (cDNA) Synthesis Kit was employed to synthesize first-strand cDNA from the collected RNA samples, adhering to the manufacturer’s guidelines. In a brief overview, the process commenced by mixing 1 μl of extracted RNA (30 μg) with 1 μl of random-hexamer primer and 6 μl of RNase-free H_2_O. Following this, the solution underwent a 5-minute incubation period at 65 °C. The microtubes were placed on ice, and each sample received 1 μl of reverse transcriptase, 1 μl of RNase inhibitor, 2 μl of dNTP mix, and 4 μl of reaction buffer. Later, the obtained solutions were rapidly subjected to incubation at 25 °C for five minutes, followed by incubation at 42 °C for 60 min. The final step involved terminating the reaction by heating at 70 °C for five minutes. Each tube contained a final volume of 20 μl for the reverse transcription process. The examination of gene expressions was carried out using a LightCycler-96 Roche device. Producing forward and reverse primers for the genes involved utilizing the custom oligonucleotide synthesis service Metabion (Martinsried, Germany). Primer-3 software was employed to design primers (refer to Table 1), and an examination of their specificity was carried out through analysis using the Basic Local Alignment Search Tool on the NCBI website (http://www.ncbi.nlm.nih.gov/tools/primer-blast/). The amplified product’s purity was affirmed via a melting curve analysis, which was conducted to validate the specific PCR product’s identity by observing the behavior of DNA strands as temperature increases. Quantifying the target mRNAs’ relative levels was achieved through the Livak method, and these levels were then standardized by comparing them to the transcript levels of the housekeeping gene glyceraldehyde 3-phosphate dehydrogenase (GAPDH). 

### Evaluation of Inflammatory Cytokine Levels

The Enzyme-Linked Immunosorbent Assay (ELISA) technique, employing commercial diagnostic kits (MyBioSource, USA), was utilized to identify and quantify the levels of tumor necrosis factor-alpha (TNF-α), interleukin-1beta (IL-1β), and interleukin-6 (IL-6) within brain homogenates. This process adhered to manufacturer-provided protocols and yielded measurements reported in the unit of pg/mg.

### Statistical analysis

The results were compared statistically through a one-way analysis of variance (ANOVA), followed by a *post-hoc* Tukey test using SPSS software version 20.0 (SPSS, Inc., Chicago, IL, USA). The data were expressed as means along with their corresponding standard deviation (mean ± SD). A single point represented each individual experimental animal. A significance level below 0.05 (*P*<0.0*5*) indicated a significant difference.

## Results

### Effects of mitotherapy on the MSS in septic rats

**Figure 1:**
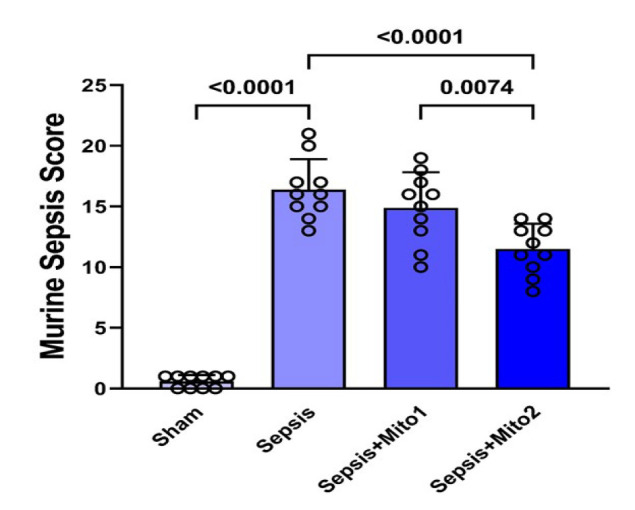
Mitotherapy decreased MSS in septic rats

The study’s findings revealed a substantial elevation in the MSS within the Sepsis group compared to the Sham group (*P*<0.00001). Administering mitochondrial transplantation through a single injection after CLP surgery showed a trend toward reducing the MSS compared to the Sepsis group. Statistical analysis demonstrated a significant reduction in the MSS for rats that underwent mitochondrial transplantation via two repetitive injections, markedly differentiating them from the Sepsis group (*P*<0.00001) and the Sepsis+Mito1 group (*P*=0.0074). However, despite the therapeutic effects, the MSS in the Sepsis+Mito2 group remained significantly elevated compared to the Sham group, indicating that mitochondrial transplantation did not lead to complete recovery within the evaluated timeframe (Figure 1).

### Effects of mitotherapy on histological changes in the brains of rats following sepsis

**Figure 2:**
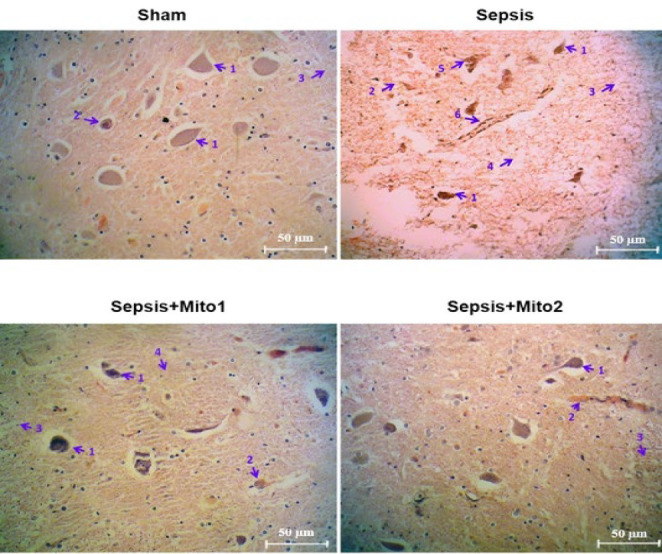
Mitotherapy improved histopathological changes in the brains of septic rats

Figure 2 illustrates the brain histopathological alterations evaluated through Hematoxylin and Eosin staining in different groups, while Table 2 summarizes the degree of brain histological changes, including the destructive architecture of myelination, endothelial cell and vascular wall damage, cytoplasmic vacuolization, and the density of Nissl bodies in pyramidal neurons across the experimental groups. In the Sham group, the brain tissue exhibited normal histological architecture, with intact white matter, well-preserved endothelial cells and vascular walls, and no signs of cytoplasmic vacuolization. Pyramidal neurons displayed normal morphology, characterized by cytoplasm rich in Nissl bodies, reflecting healthy and intact brain tissue. In the Sepsis group, prominent pathological changes were observed, including the destructive architecture of myelination, significant endothelial cell and vascular wall damage, and pronounced cytoplasmic vacuolization. Additionally, many pyramidal neurons exhibited cytoplasm devoid of Nissl bodies, indicating severe neuronal injury. Mitochondrial transplantation via a single injection resulted in partial recovery from sepsis-induced brain injury. The destructive architecture of myelination was less severe compared to the Sepsis group, and there was some recovery in endothelial cell and vascular wall damage. Cytoplasmic vacuolization decreased to a moderate level, and the density of Nissl bodies in pyramidal neurons increased, although it remained lower than that observed in the Sham group. In contrast, two repetitive injections of isolated mitochondria led to the most significant improvement, approaching Sham group levels. The destructive architecture of myelination was significantly reduced, with endothelial cell and vascular wall damage being mild relative to the Sepsis group. Furthermore, cytoplasmic vacuolization was further diminished, while the density of Nissl bodies in pyramidal neurons exhibited a marked increase.

### Effects of mitotherapy on mitochondrial functional indices in the brains of rats following sepsis

**Figure 3:**
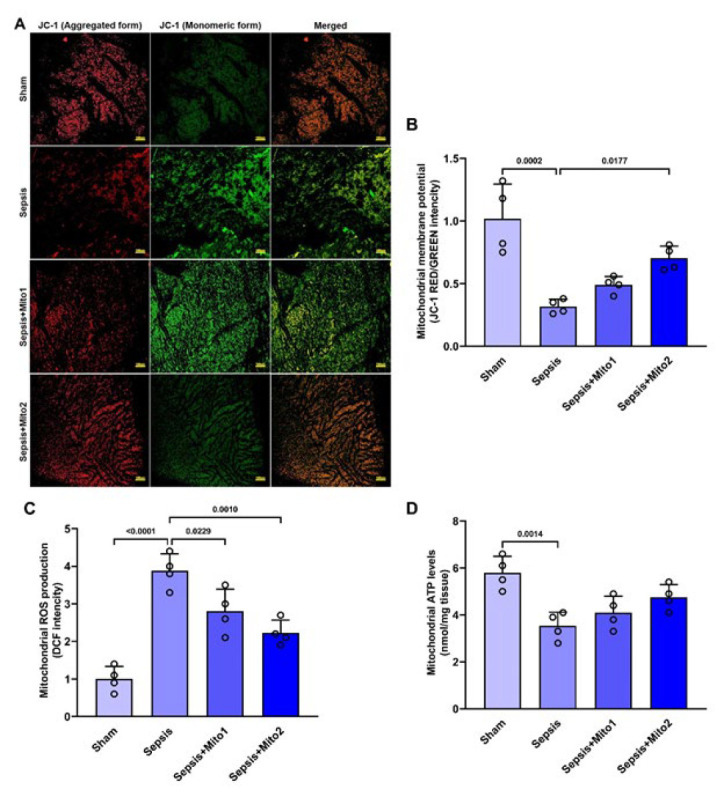
Mitotherapy improved mitochondrial functional indices in the brains of septic rats

The variations in three key aspects of mitochondrial function (mitochondrial membrane potential, ROS production, and ATP content) are depicted in Figure 3A-D. In comparison to those observed in the Sham group, induction of sepsis through CLP led to a significant reduction in the red/green fluorescence intensity ratio (*P*=0.0002; Figure 3B), an increase in ROS production (*P*<0.00001; Figure 3C), and a decrease in ATP content (*P*=0.0014; Figure 3D). Treatment with a single injection of isolated mitochondria post-CLP resulted in a notable decrease in ROS production compared to the Sepsis group (*P*=0.0229; Figure 3C). However, no changes were observed in the red/green fluorescence intensity ratio (*P*=0.4163; Figure 3B) or ATP content (*P*=0.5928; Figure 3D). In contrast, treatment with repetitive injections of isolated mitochondria post-CLP displayed a substantial increase in the red/green fluorescence intensity ratio (*P*=0.0177 vs Sepsis group; Figure 3B) and a notable reduction in ROS production (*P*=0.*0010* vs Sepsis group; Figure 3C), nevertheless, ATP content remained unchanged within this group (*P*=0.0761 vs Sepsis group; Figure 3D). Despite these improvements, it is important to note that mitochondrial functional indices in the treatment groups did not fully recover to Sham levels (Figure 3B, C).

### Effects of mitotherapy on the expression of genes regulating mitochondrial biogenesis and dynamics in the brains of rats following sepsis

**Figure 4:**
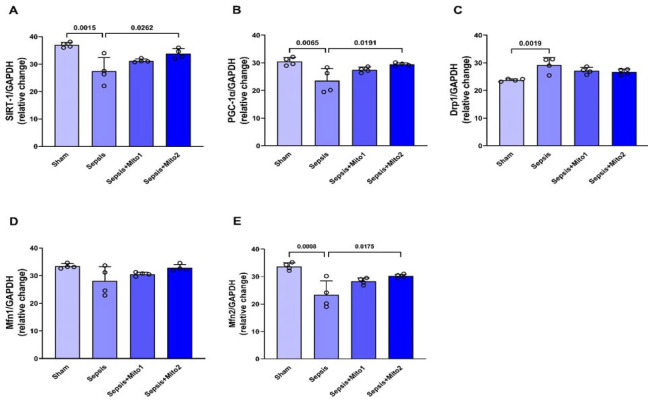
Mitotherapy improved the expression of genes related to mitochondrial biogenesis and dynamics in the brains of septic rats

Figure 4A-E illustrates the expression of genes involved in mitochondrial biogenesis (SIRT-1 and PGC-1α), fission (Drp1), and fusion (Mfn1 and Mfn2) in rat brains. In comparison to the Sham group, septic rats exhibited a reduction in the gene expression levels of SIRT-1 (*P*=0.0015; Figure 4A), PGC-1α (*P*=0.0065; Figure 4B), and Mfn2 (*P*=0.0008; Figure 4E), while the expression of Drp1 gene increased (*P*=0.0019; Figure 4C). Mitotherapy one hour after CLP appeared to reverse the changes in gene expression caused by CLP partially; however, these changes did not achieve statistical significance (Figure 4A-E). Mitotherapy involving repetitive injections after CLP led to a restoration of gene expression levels of SIRT-1 (*P*=0.0262 vs Sepsis group), PGC-1α (*P*=0.0191 vs Sepsis group), and Mfn2 (*P*=0.0175 vs Sepsis group), bringing them closer to the levels observed in the pre-damage state (Figure 4A, B, E). However, it did not significantly affect the expression of Drp1 and Mfn1 genes (Figure 4C, D). No significant differences in Mfn1 expression were observed across the experimental groups (Figure 4D). 

### Effect of mitotherapy on inflammatory cytokine levels in the brains of rats following sepsis

**Figure 5:**
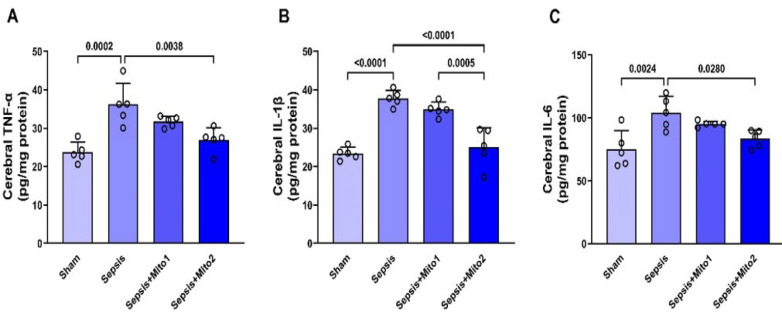
Mitotherapy alleviated inflammatory cytokine levels in the brains of septic rats

The investigation into inflammatory cytokine levels in the brains of rats subjected to CLP revealed higher concentrations of TNF-α, IL-1β, and IL-6 when compared to the Sham group (*P*=0.0002, *P<*0.00001, and *P*=0.0024, respectively) (Figure 5A-C). Mitochondrial transplantation conducted one hour after CLP showed a tendency towards reducing TNF-α, IL-1β, and IL-6 levels compared to the Sepsis group. Nevertheless, these observed alterations did not achieve statistical significance (*P*=0.2267, *P*=0.4394, and *P*=0.5457, respectively) (Figure 5A-C). In the group that received two repetitive injections of isolated mitochondria after CLP, there was a notable decrease in TNF-α, IL-1β, and IL-6 levels. These reductions were found to be statistically significant when compared to the Sepsis group (*P*=0.*0038*, *P*<0.00001, and *P*=0.0280, respectively) (Figure 5A-C). Additionally, mitotherapy involving repetitive injections after CLP resulted in a significant decrease in the IL-1β level when compared to the Sepsis+Mito1 group (*P*=0.0005) (Figure 5B). However, no significant changes were observed in the levels of TNF-α (*P*=0.1829) and IL-6 (*P*=0.3062) between these groups (Figures 5A and C).

## Discussion

This experimental study provided evidence of the positive impact of mitotherapy in alleviating sepsis-induced brain injury in rats. Mitotherapy at one hour after CLP demonstrated partial neuroprotective effects. It is worth noting that mitotherapy, administered intravenously both at the one-hour and seven-hour marks after CLP, yielded remarkable neuroprotective effects against sepsis-induced brain injury. These effects were validated by significant improvements in the behavioral phenotype and brain histopathological changes following CLP-induced sepsis. The results of our study offer compelling evidence to support the notion that the neuroprotective effects of mitotherapy are intricately linked with the improvement of mitochondrial function. This improvement aligns with increased expression of genes regulating mitochondrial biogenesis (SIRT-1 and PGC-1α), the promotion of the Mfn2 gene associated with mitochondrial fusion, and suppressing inflammatory response following CLP-induced sepsis.

Previous works have shown that mitochondrial biogenesis becomes disrupted upon septic challenge, which can lead to the release of damage-associated molecular patterns (DAMPs) and ROS, initiating immune cell activation and furthering the spread of inflammation (26-30). Besides, augmented fission and reduced fusion dynamics lead to mitochondrial fragmentation and impaired mitochondrial connectivity, respectively (31-33). Dysfunctional mitochondria release ROS and DAMPs, amplifying the inflammatory signal and propagating tissue damage. Simultaneously, inflammatory cytokines can impair mitochondrial function and disrupt energy metabolism (34, 35). Our study, in line with previous reports, indicated a correlation between sepsis-induced brain injury and elevated levels of inflammatory cytokines, pointing to an exacerbated inflammatory response in sepsis. In addition to the inflammatory aspect, our study sheds light on mitochondrial dysfunction as a significant player in sepsis-induced brain injury. Specifically, disruptions in mitochondrial biogenesis and fission/fusion dynamics were evident. The current work confirmed that mitotherapy significantly improved both the behavioral phenotype and brain histopathological changes in the context of sepsis. These beneficial effects of mitotherapy were paralleled by the restoration of mitochondrial function. Interestingly, this work unveiled an even more robust effect when mitotherapy was administered through two repetitive injections. The enhanced potency of repetitive injections in mitigating brain mitochondrial dysfunction following sepsis arises from their ability to provide sustained and cumulative mitochondrial support. The sustained presence of exogenous mitochondria likely stimulated a greater degree of mitochondrial biogenesis. This augmented biogenesis may have additive effects with the introduced mitochondria, further enhancing overall mitochondrial function. Furthermore, the elevated expression of the mitochondrial fusion gene, Mfn2, indicates improved mitochondrial network dynamics. This dynamic interconnectedness may facilitate the maintenance of mitochondrial health, contributing to enhanced cellular energy production and stress mitigation. Besides, the sustained presence of functional mitochondria may modulate inflammatory pathways and reduce the release of pro-inflammatory cytokines. This cumulative effect may contribute to minimizing the inflammatory burden on brain tissue and promoting overall tissue recovery. 

To reinforce our conclusions, several studies have highlighted the neuroprotective effects of mitotherapy, achieved through various mechanisms. *In vivo* and *in vitro* studies by Yan *et al*. provided evidence that mitochondrial transplantation exhibited neuroprotective properties against sepsis-associated brain dysfunction by influencing microglial polarization and reducing the inflammatory response (16). In a mouse stroke model, mitotherapy has shown promising results in decreasing mortality and enhancing emotional and cognitive function by reducing the infarct area, suppressing pyroptosis, and stimulating neurogenesis in the ischemic cortex. It was also suggested that S100 calcium-binding protein A9, a main downstream component of the interleukin-17 pathway, might play a role in enhancing the anti-pyroptotic and pro-neurogenic effects of exogenous mitochondria by increasing their uptake by microglia and fusion with endogenous mitochondria (36). A study by Zhang *et al*. revealed that administering mitochondria via the lateral ventricles led to a neuroprotective effect following cerebral ischemia/reperfusion injury by reducing cellular oxidative stress and apoptosis (37). Another investigation documented restoring brain tissue damage induced by ischemia/reperfusion using mitochondria derived from mesenchymal stem cells. This restoration was linked to the inhibition of apoptosis and microglial activation, thereby averting the formation of brain tissue scar indicated by reduced astrogliosis (38). Additionally, mitochondrial transplantation represents an innovative therapeutic strategy for protecting neuronal cells against ferroptotic cell death. Chen *et al*. found that isolated exogenous mitochondria incorporated into both healthy and ferroptotic neuronal cells lead to increased metabolic activity and cell survival by reducing lipid peroxidation and mitochondrial superoxide production. The neuroprotective effects were attributed to the activities of mitochondrial complexes I, III, and V. Likewise, the internalization of exogenous mitochondria in mouse primary cortical neurons preserved neuronal networks when exposed to ferroptotic stimuli (39). Our study aligns with these findings, further validating the potential of mitotherapy as a therapeutic intervention for sepsis-associated brain injury.

Delivering isolated mitochondria to the brain is still a challenge, affecting both the mitochondria’s distribution within cells and their therapeutic efficacy. According to previous reports, transplanted mitochondria were found in brain resident cells after intracerebral injection (38, 40). However, the invasive nature of intracerebral injection limits the translational relevance of these findings to the patient’s benefit, as it may result in tissue damage. Moreover, sepsis often leads to widespread brain injury rather than isolated lesions localized to specific areas or circuits (12, 41). Intravenous administration has emerged as a less invasive and technically simpler method for delivering mitochondria, allowing for easier repeat dosing if necessary. This method offers a more clinically relevant approach for delivering mitochondria to the septic brain, where the exact injury site may not be known. While the intact BBB typically restricts the passage of infused mitochondria, a considerable number of mitochondria can traverse the BBB following its disruption in various central nervous system (CNS) disorders such as cerebral ischemia/reperfusion injury (42, 43), Alzheimer’s disease (44), and Parkinson’s disease (45). Occasionally, these mitochondria incorporate into neurons and other CNS cells. In sepsis scenarios, disruption of BBB integrity also occurs, allowing isolated mitochondria to pass from the bloodstream into the brain (30). In light of the evidence from previous studies (42-45), it is worth mentioning that our study also revealed the neuroprotective effects of mitotherapy following intravenous injection post-CLP. These effects correlated with enhanced mitochondrial function and suppression of inflammatory response in the septic brain tissue, reflecting that transplanted mitochondria were present in brain resident cells, which induced neuroprotection. Histopathological analysis showed the neuroprotective effects exerted by these mitochondria. While the effects on histopathology are likely direct, it is important to note that the evidence is currently insufficient to determine that mitotherapy directly caused the observed cytokine reduction conclusively. It is plausible that other mechanisms and signaling pathways are involved in modulating cytokine levels. Further studies are required to validate and delve deeper into the potential therapeutic effects of intravenous delivery of isolated mitochondria in the context of sepsis-induced brain injury. As a final point, elevated blood calcium levels during sepsis can lead to the opening of mitochondrial permeability transition pores in isolated mitochondria. This can subsequently result in a decline in their viability when introduced into the systemic circulation via intravenous injection (46). Using agents to enhance their survival in the systemic circulation and facilitate their transfer and uptake in the brain after intravenous injection can boost the therapeutic effectiveness of mitotherapy (47).

### Limitations and suggestions 

While this study provides promising evidence for the neuroprotective effects of mitotherapy, several limitations should be addressed in future research. We did not extensively characterize the mitochondrial cargo transferred during transplantation or assess the functional status of the transplanted mitochondria. Future studies should explore these aspects to better understand their role in therapeutic outcomes. It is important to note that our study specifically targeted the prefrontal cortex. Future research should evaluate the effects of mitotherapy on spatial learning and memory, which are primarily linked to the hippocampus. Additionally, while we assessed key mitochondrial functional endpoints, future studies should include more comprehensive evaluations, such as oxidative phosphorylation capacity, respiratory chain complex activity, and mitochondrial DNA content, to better understand the impact of mitochondrial transplantation. Evaluating the expression of proteins involved in mitochondrial biogenesis and dynamics would also provide a clearer understanding of the functional impact of mitotherapy. Our study primarily assessed outcomes at a 24-hr post-surgery time point, and longer-term studies are needed to determine the sustainability of the observed effects and their implications for recovery. We also acknowledge the need to explore the optimal therapeutic window and dosage of mitochondrial injections. Investigating these factors will help refine treatment protocols and enhance therapeutic outcomes. Intravenous administration, as used in our study, offers a minimally invasive approach for systemic mitochondrial delivery, making it well-suited for treating diffuse damage like sepsis-induced brain injury. Although challenges such as tissue-specific targeting and mitochondrial degradation persist, advancements in labeling, nanoparticle-based delivery, and molecular engineering hold promise. Future research should incorporate advanced imaging to confirm mitochondrial biodistribution and integration and electrophysiological experiments to assess their impact on neuronal activity and synaptic function. 

MSS in different groups. The data were expressed as mean ± standard deviation (n = 10 for each group). Sepsis+Mito1: Septic rats receiving mitotherapy one hour following cecal ligation and puncture surgery; Sepsis+Mito2: Septic rats receiving mitotherapy one hour and seven hours following cecal ligation and puncture surgery.

Brain histopathological evaluation through light microscopy using Hematoxylin and Eosin staining in different groups (n = 4 for each group). Sham Group: Pyramidal neuron with a normal shape and cytoplasm containing distinct Nissl bodies (1); pyramidal neuron with a clear nucleus (2); normal architecture of myelination (3). Sepsis Group: Dense and pyknotic nucleus, along with cytoplasmic vacuolization (1); reduced size of pyramidal neuron (2); destructive architecture of myelination (3); gray matter deformity and neuronal destruction (4); pyramidal neuron with cytoplasm lacking Nissl bodies (5); increased vascular lumen and discontinuity of the vascular wall (6). Sepsis+Mito1 Group: Partial damage to pyramidal neuron, including decreased size, condensed nucleus, and reduced accumulation of Nissl bodies (1); blood vessel without proliferation and endothelial separation (2); normal architecture of myelination (3); uniformity of gray matter homogeneity (4). Sepsis+Mito2 Group: Pyramidal neuron with a normal shape, clear nucleus, and cytoplasm containing distinct Nissl bodies (1); blood vessels without proliferation and endothelial separation (2); normal architecture of myelination (3). Sepsis+Mito1: Septic rats receiving mitotherapy one hour following cecal ligation and puncture surgery; Sepsis+Mito2: Septic rats receiving mitotherapy one hour and seven hours following cecal ligation and puncture surgery.

Representative fluorescent images of JC-1 staining in brain tissue from different groups (magnification: × 40). The aggregated red fluorescent signal and the monomeric green fluorescent signal were detected using a fluorescent microscope. The merged photos were used to assess mitochondrial membrane potential by calculating the ratio of red to green fluorescent intensities (A); Mitochondrial membrane potential alterations (B); Reactive oxygen species (ROS) production (C); and adenosine triphosphate (ATP) content (D) in different groups. The data were expressed as mean ± standard deviation (n = 4 for each group). Sepsis+Mito1: Septic rats receiving mitotherapy one hour following cecal ligation and puncture surgery; Sepsis+Mito2: Septic rats receiving mitotherapy one hour and seven hours following cecal ligation and puncture surgery.

The expression of sirtuin 1 (SIRT-1) (A); Peroxisome proliferator-activated receptor gamma coactivator 1-alpha (PGC-1α) (B); Dynamin-related protein-1 (Drp1) (C); Mitofusin 1 (Mfn1) (D); and mitofusin 2 (Mfn2) (E) genes in different groups. The data were expressed as mean ± standard deviation (n = 4 for each group). Sepsis+Mito1: Septic rats receiving mitotherapy one hour following cecal ligation and puncture surgery; Sepsis+Mito2: Septic rats receiving mitotherapy one hour and seven hours following cecal ligation and puncture surgery.

The levels of tumor necrosis factor-alpha (TNF-α) (A); Interleukin-1β (IL-1β) (B); and (C) Interleukin-6 (IL-6) (B) in different groups. The data were expressed as mean ± standard deviation (n = 5 for each group). Sepsis+Mito1: Septic rats receiving mitotherapy one-hour following cecal ligation and puncture surgery; Sepsis+Mito2: Septic rats receiving mitotherapy one hour and seven hours following cecal ligation and puncture surgery.

## Conclusion

This study offers an interesting viewpoint by highlighting the potential of mitotherapy, especially through repetitive injections, to bring about neuroprotection in a rat model of CLP-induced sepsis. The neuroprotective benefits of mitotherapy were linked to the restoration of mitochondrial function, up-regulation of mitochondrial biogenesis and fusion genes, and reduction of inflammatory cytokine levels. It is worth noting that the mediating function of the SIRT-1/PGC-1α network in these beneficial mechanisms was also observed. The efficacy of repetitive mitochondrial injections at distinct time points post-CLP suggests the importance of timing in optimizing therapeutic outcomes. These findings open avenues for further research into tailored mitotherapy protocols and exploration of its potential in sepsis management.

## Data Availability

The datasets used in this study are available from the corresponding authors upon reasonable request.

## Declaration

The authors declare that they have not used any AI tools in the preparation of this manuscript.

## Fuding

The Drug Applied Research Center and the Vice Chancellor for Research at Tabriz University of Medical Sciences, Tabriz, Iran, financially supported the research, authorship, and publication of this article.

## References

[1] Arora J, Mendelson AA, Fox-Robichaud A (2023). Sepsis: Network pathophysiology and implications for early diagnosis. Am J Physiol Regul Integr Comp Physiol.

[2] Sonneville R, Benghanem S, Jeantin L, de Montmollin E, Doman M, Gaudemer A (2023). The spectrum of sepsis-associated encephalopathy: A clinical perspective. Crit Care.

[3] Catarina AV, Branchini G, Bettoni L, De Oliveira JR, Nunes FB (2021). Sepsis-associated encephalopathy: From pathophysiology to progress in experimental studies. Mol Neurobiol.

[4] Zhao L, Gao Y, Guo S, Lu X, Yu S, Ge ZZ (2021). Sepsis-Associated encephalopathy: Insight into injury and pathogenesis. CNS Neurol Disord Drug Targets.

[5] Xin Y, Tian M, Deng S, Li J, Yang M, Gao J (2023). The key drivers of brain injury by systemic inflammatory responses after sepsis: Microglia and neuroinflammation. Mol Neurobiol.

[6] Manfredini A, Constantino L, Pinto MC, Michels M, Burger H, Kist LW (2019). Mitochondrial dysfunction is associated with long-term cognitive impairment in an animal sepsis model. Clin Sci.

[7] Wu Y, Yao Y-M, Lu Z-Q (2019). Mitochondrial quality control mechanisms as potential therapeutic targets in sepsis-induced multiple organ failure. J Mol Med.

[8] Pan S, Lv Z, Wang R, Shu H, Yuan S, Yu Y (2022). Sepsis-induced brain dysfunction: pathogenesis, diagnosis, and treatment. Oxid Med Cell Longev.

[9] van der Slikke EC, Star BS, Quinten VM, Ter Maaten JC, Ligtenberg JJ, van Meurs M (2022). Association between oxidized nucleobases and mitochondrial DNA damage with long-term mortality in patients with sepsis. Free Radic Biol Med.

[10] Montero-Jodra A, de la Fuente MÁ, Gobelli D, Martín-Fernández M, Villar J, Tamayo E (2024). The mitochondrial signature of cultured endothelial cells in sepsis: Identifying potential targets for treatment. Biochim Biophys Acta Mol Basis Dis.

[11] Zhang H, Feng Y-w, Yao Y-m (2018). Potential therapy strategy: Targeting mitochondrial dysfunction in sepsis. Mil Med Res.

[12] Arina P, Singer M (2021). Pathophysiology of sepsis. Curr Opin Anesthesiol.

[13] Huang Y, Chen R, Jiang L, Li S, Xue Y (2021). Basic research and clinical progress of sepsis-associated encephalopathy. J Intensive Care Med.

[14] Mokhtari B, Yavari R, Badalzadeh R, Mahmoodpoor A (2022). An overview on mitochondrial-based therapies in sepsis-related myocardial dysfunction: Mitochondrial transplantation as a promising approach. Can J Infect Dis Med Microbiol.

[15] Park A, Oh M, Lee SJ, Oh K-J, Lee E-W, Lee SC (2021). Mitochondrial transplantation as a novel therapeutic strategy for mitochondrial diseases. Int J Mol Sci.

[16] Yan C, Ma Z, Ma H, Li Q, Zhai Q, Jiang T (2020). Mitochondrial transplantation attenuates brain dysfunction in sepsis by driving microglial M2 polarization. Mol Neurobiol.

[17] Hwang JW, Lee MJ, Chung TN, Lee HAR, Lee JH, Choi SY (2021). The immune modulatory effects of mitochondrial transplantation on cecal slurry model in rat. Crit Care.

[18] Zhang Z, Yan C, Miao J, Pu K, Ma H, Wang Q (2021). Muscle-derived mitochondrial transplantation reduces inflammation, enhances bacterial clearance, and improves survival in sepsis. Shock.

[19] Mokhtari B, Hamidi M, Badalzadeh R, Mahmoodpoor A (2023). Mitochondrial transplantation protects against sepsis-induced myocardial dysfunction by modulating mitochondrial biogenesis and fission/fusion and inflammatory response. Mol Biol Rep.

[20] Bafadam S, Mokhtari B, Vafaee MS, Oscuyi ZZ, Nemati S, Badalzadeh R (2024). Mitochondrial transplantation combined with coenzyme Q10 induces cardioprotection and mitochondrial improvement in aged male rats with reperfusion injury. Exp Physiol.

[21] Preble JM, Pacak CA, Kondo H, MacKay AA, Cowan DB, McCully JD (2014). Rapid isolation and purification of mitochondria for transplantation by tissue dissociation and differential filtration. J Vis Exp.

[22] Rittirsch D, Huber-Lang MS, Flierl MA, Ward PA (2009). Immunodesign of experimental sepsis by cecal ligation and puncture. Nat Protoc.

[23] Tuon L, Comim CM, Petronilho F, Barichello T, Izquierdo I, Quevedo J (2008). Time-dependent behavioral recovery after sepsis in rats. Intensive Care Med.

[24] Shrum B, Anantha RV, Xu SX, Donnelly M, Haeryfar SM, McCormick JK (2014). A robust scoring system to evaluate sepsis severity in an animal model. BMC Res Notes.

[25] Cardiff RD, Miller CH, Munn RJ (2014). Manual hematoxylin and eosin staining of mouse tissue sections. Cold Spring Harb Protoc.

[26] Zhu Y, Wang K, Ma Z, Liu D, Yang Y, Sun M (2019). SIRT1 activation by butein attenuates sepsis-induced brain injury in mice subjected to cecal ligation and puncture via alleviating inflammatory and oxidative stress. Toxicol Appl Pharmacol.

[27] Xie K, Wang Y, Yin L, Wang Y, Chen H, Mao X (2021). Hydrogen gas alleviates sepsis-induced brain injury by improving mitochondrial biogenesis through the activation of PGC-α in mice. Shock.

[29] Zhou M, Aziz M, Wang P (2021). Damage-associated molecular patterns as double-edged swords in sepsis. Antioxid Redox Signal.

[30] Haileselassie B, Joshi AU, Minhas PS, Mukherjee R, Andreasson KI, Mochly-Rosen D (2020). Mitochondrial dysfunction mediated through dynamin-related protein 1 (Drp1) propagates impairment in blood brain barrier in septic encephalopathy. J Neuroinflammation.

[31] Deng S, Ai Y, Gong H, Feng Q, Li X, Chen C (2018). Mitochondrial dynamics and protective effects of a mitochondrial division inhibitor, Mdivi-1, in lipopolysaccharide-induced brain damage. Biochem Biophys Res Commun.

[32] Zhu Y, Kuang L, Wu Y, Deng H, She H, Zhou Y (2021). Protective effects of inhibition of mitochondrial fission on organ function after sepsis. Front Pharmacol.

[33] Suliman HB, Piantadosi CA (2016). Mitochondrial quality control as a therapeutic target. Pharmacol Rev.

[34] Nagar H, Piao S, Kim C-S (2018). Role of mitochondrial oxidative stress in sepsis. Acute Crit Care.

[35] Kong C, Song W, Fu T (2022). Systemic inflammatory response syndrome is triggered by mitochondrial damage. Mol Med Rep.

[36] Sun L, Zhao Z, Guo J, Qin Y, Yu Q, Shi X (2024). Mitochondrial transplantation confers protection against the effects of ischemic stroke by repressing microglial pyroptosis and promoting neurogenesis. Neural Regen Res.

[37] Zhang Z, Ma Z, Yan C, Pu K, Wu M, Bai J (2019). Muscle-derived autologous mitochondrial transplantation: A novel strategy for treating cerebral ischemic injury. Behav Brain Res.

[38] Pourmohammadi-Bejarpasi Z, Roushandeh AM, Saberi A, Rostami MK, Toosi SMR, Jahanian-Najafabadi A (2020). Mesenchymal stem cells-derived mitochondria transplantation mitigates I/R-induced injury, abolishes I/R-induced apoptosis, and restores motor function in acute ischemia stroke rat model. Brain Res Bull.

[39] Chen T, Majerníková Na, Marmolejo-Garza A, Trombetta-Lima M, Sabogal-Guáqueta AM, Zhang Y (2023). Mitochondrial transplantation rescues neuronal cells from ferroptosis. Free Radic Biol Med.

[40] Zhao J, Qu D, Xi Z, Huan Y, Zhang K, Yu C (2021). Mitochondria transplantation protects traumatic brain injury via promoting neuronal survival and astrocytic BDNF. Transl Res.

[41] Sekino N, Selim M, Shehadah A (2022). Sepsis-associated brain injury: Underlying mechanisms and potential therapeutic strategies for acute and long-term cognitive impairments. J Neuroinflammation.

[42] Norat P, Sokolowski JD, Gorick CM, Soldozy S, Kumar JS, Chae Y (2023). Intraarterial transplantation of mitochondria after ischemic stroke reduces cerebral infarction. Stroke Vasc Interv Neurol.

[43] Nakamura Y, Lo EH, Hayakawa K (2020). Placental mitochondria therapy for cerebral ischemia-reperfusion injury in mice. Stroke.

[44] Nitzan K, Benhamron S, Valitsky M, Kesner EE, Lichtenstein M, Ben-Zvi A (2019). Mitochondrial transfer ameliorates cognitive deficits, neuronal loss, and gliosis in Alzheimer’s disease mice. J Alzheimer’s Dis.

[45] Shi X, Zhao M, Fu C, Fu A (2017). Intravenous administration of mitochondria for treating experimental Parkinson’s disease. Mitochondrion.

[46] Bertero E, Maack C, O’Rourke B (2018). Mitochondrial transplantation in humans:“magical” cure or cause for concern?. J Clin Investig.

[47] Chang J-C, Wu S-L, Liu K-H, Chen Y-H, Chuang C-S, Cheng F-C (2016). Allogeneic/xenogeneic transplantation of peptide-labeled mitochondria in Parkinson’s disease: Restoration of mitochondria functions and attenuation of 6-hydroxydopamine–induced neurotoxicity. Transl Res.

